# Gender Differences in Depressive Traits among Rural and Urban Chinese Adolescent Students: Secondary Data Analysis of Nationwide Survey CFPS

**DOI:** 10.3390/ijerph18179124

**Published:** 2021-08-30

**Authors:** Weilong Chen, Yi Huang, Abanoub Riad

**Affiliations:** 1 Guang’an Vocational and Technical College, Guang’an 638000, China; chenweilong@126.com; 2 Department of Psychology, Faculty of Social Studies, Masaryk University, Joštova 218, 602 00 Brno, Czech Republic; 3 Institute for Research of Children, Youth and Family, Faculty of Social Studies, Masaryk University, Joštova 218, 602 00 Brno, Czech Republic; 4 Department of Public Health, Faculty of Medicine, Masaryk University, Kamenice 5, 625 00 Brno, Czech Republic; abanoub.riad@med.muni.cz; 5 Czech National Centre for Evidence-Based Healthcare and Knowledge Translation (Cochrane Czech Republic, Czech EBHC: JBI Centre of Excellence, Masaryk University GRADE Centre), Institute of Biostatistics and Analyses, Faculty of Medicine, Masaryk University, Kamenice 5, 625 00 Brno, Czech Republic

**Keywords:** adolescents, China, depressive traits, gender equality, health inequalities, regional equality, socioeconomic status

## Abstract

Many previous studies have indicated that urban adolescents show a higher level of mental health in China compared to rural adolescents. Specifically, girls in rural areas represented a high-risk group prior to the 21st century, demonstrating more suicidal behaviour and ideation than those in the urban areas because of the severe gender inequality in rural China. However, because of the urbanisation process and centralised policy to eliminate gender inequality in recent decades, the regional and gender differences in mental health might decrease. This research aimed to probe the gender and regional differences in depressive traits among adolescent students currently in China. We adopted the national survey dataset Chinese Family Panel Studies (CFPS) conducted in 2018. Accordingly, 2173 observations from 10–15-year-old subjects were included. CFPS utilised an eight-item questionnaire to screen individuals’ depressive traits. Two dimensions of depressive traits were confirmed by CFA, namely depressed affect and anhedonia. The measurement invariance tests suggested that the two-factor model was applicable for both males and females and rural and urban students. Based on the extracted values from the CFA model, MANOVA results revealed that, compared to boys, girls experienced more depressed affect. Moreover, rural students demonstrated more anhedonia symptoms. There was no interaction between gender and region. The results suggest that, even though the gender and regional differences are small, being a female and coming from a rural area are still potential risk factors for developing depressive traits among adolescent students in China.

## 1. Introduction

The differences between rural and urban areas in China have been investigated for a long time. Generally, rural areas and urban areas represent different worlds in China. There are huge social inequalities across the two areas, including economic aspects, access to medical support, and educational resources [1,2,3]. Regarding the economic gap, in fact, the rural–urban gap of inequality of income has become larger since 1978 [4]. In the 1980s, the Gini coefficient was around 0.3, and by 2013, it reached the level of 0.61, compared with around 0.45 in the United States [4]. Regarding the health service aspect, according to the China Health and Nutrition Survey from 1993 to 2011, the rate of obtaining professional medical care is higher among Chinese urban citizens [3]. Regarding the gap of education between rural and urban areas of China, scholars have pointed out that the identity of the rural or urban resident plays a determining role to predict the likelihood of the transition from the compulsory education stage to senior high school [5]. Compared to urban residents, rural people demonstrate much lower satisfaction with respect to educational opportunities [6].

The school environment is strongly associated with the well-being and mental health of young people. According to ecological system theory [7], the environments children directly interact with play an important role in their cognitive and affective development. Such environments include family, school, and peer relationships. Researchers argued that school could be seen as a source to provide social support. It was found that positive teacher–student interaction and peer relationships can decrease the risk of anxiety disorder and increase the level of self-esteem, which is an important protective factor of depression [8,9]. Moreover, teacher’s professional knowledge and skills related to mental health can be transferred to students and eventually improve students’ mental health help-seeking behaviours [10].

However, there might be inequalities in school and educational environment in China between urban and rural areas, which could cause regional inequality in psychological health. A report in 2006 suggested that the teachers working in rural China have lower job satisfaction because of a lack of access to transportation, as well as cultural and educational resources, which may cause negative interaction between teachers and students [11]. Peng et al. suggested that, compared to teachers in the urban area of China, rural teachers have fewer resources to promote their professional skills as well as fewer lifelong learning opportunities, which might lead to the poorer perception of students’ mental disorders and inadequate intervention [12]. Besides, in the American context, there is difficulty in terms of access to psychiatric services for rural residents, and it is plausible that China is very likely to have the same problem, which leads to a higher prevalence of mental health problems [13]. Therefore, in conclusion, rural China is a disadvantaged environment for students’ mental health compared to urban China.

In addition to location, for adolescents, gender is another demographic factor that might affect mental health. However, it is worth noticing that, regarding one of the typical mental health problems, depression, the gender difference during the adolescent stage is a complicated issue. On the one hand, some theories have argued that females suffer more from depression than males. First, according to biopsychology, because of their genetic vulnerability and levels of cortisol, females respond to stress more negatively [14,15]. Second, from the perspective of pure psychological factors, the traditional gender concept raises the expectation that males should demonstrate greater individualism and assertiveness while females are emotional and sensitive. Thus, under such a rearing context, when facing stress events, males are more easily to develop problematic behaviours (such as addiction to alcohol, drugs use) and females are more likely to generate internalising disorders [15]. Nevertheless, on the other hand, because of the stereotype of gender differences, male adolescents, especially early adolescents, have a stronger tendency to refuse to seek social support and psychiatric help to relieve their stress, even when professional psychological service and social support are provided [16,17]. At the same time, boys have less mental health knowledge, which is a typical mental health risk. Moreover, boys demonstrate a higher mental health stigma, which hinders them from seeking support against their mental stress [16].

In fact, in the Chinese context, there might be an interaction between gender and region in adolescents’ mental health. In the 1990s, it was found that the gender inequality in mental health was much stronger in rural China, particularly among the youth [18,19,20]. Even for those in early adulthood, advanced educated university students living in city areas did not demonstrate the gender differences in depression [21]. The differences in the degree of inequality among regions (rural area vs urban area) can be explained by the social expectations and cultural norms. In urban China during the 1990s, because of the advocacy of gender equality by the centralised policy, females were encouraged to be independent and chase advanced education. These political efforts eliminated the gender inequality in mental health to some extent [22,23]. However, due to the huge gap between cities and the countryside, in rural China during that time, the traditional ideology for females was still the mainstream. Young females were seen as “producers of sons” in male-dominated families instead of independent individuals with the right to pursue their own self-value. Thus, rural females faced great educational deprivation at the same time [24,25]. According to Oakley’s gender socialisation theory, gender is a concept determined by environment and culture via verbal and nonverbal signifiers (such as interpersonal relationships, media use), social value and belief, and stereotypes [26,27]. Therefore, theoretically, due to the different gender roles for females in rural and urban China in the 1990s, it is reasonable to find the distinction in female’s self-esteem and mental health between rural and urban areas.

Depression is one of the common mental health problems among adolescents [28,29,30]. The global trend of adolescent depression has increased over the years. According to a Chinese study covering eight large cities, the rate of weekly experience of depressive symptoms among adolescents was 44.2%, much higher than the findings from abroad [31,32].

This study aimed to investigate the gender differences in depressive traits in the context of education location (rural area vs. urban area). We utilised Chinese national survey data collected in 2018. If the gap of social inequalities between rural and urban areas is remains significant, it is plausible that, overall, the depressive traits among adolescent students in rural schools are more severe than students in cities. In addition, it is valuable to exam the achievement of China’s efforts in promoting compulsory education for both genders and the protection of women’s rights, especially for rural China. If the progress has been slight over the years, the pattern of gender differences in depressive traits would remain consistent with the 1990, i.e., in rural areas, the rate of mental health problems of females would be greater in comparison to urban areas.

## 2. Materials and Methods

### 2.1. Data Sources

We adopted the data of the Chinese Family Panel Survey (CFPS) from 2018. CFPS is a national longitudinal program initiated in 2010, collecting data from 25 provinces of China covering 95% of the Chinese population. The sampling method of CFPS is based on the multi-stage approach [33]. CFPS program collects data every two years, and it aims to investigate families’ and individuals’ information on a range of topics, including economic status, educational background, work status, as well as physical and mental health.

This study used the data collected in 2018, which was the newest data we could obtain. According to the CFPS’s definition of adolescents, we included observations from 10–15 year-old adolescents who had adequate cognitive ability to finish self-reported questionnaires. After removing cases with missing values, there were 2173 adolescents in this study.

### 2.2. Ethical Considerations

CFPS is a public database. Therefore, ethics approval and consent to participate were not applicable in this study. The data collection work of CFPS had been already approved by the Ethics Committees of the Institution of Social Science Survey, Peking University.

### 2.3. Measurements

#### 2.3.1. Depressive Traits

CFPS-2018 employed a special 8-item scale to screen individuals’ depressive traits [34]. According to the CFPS manual, this scale was designed for individuals with sufficient language and logical thinking capability. People with too severe cognitive or physical disorders to answer the questionnaire should not be included in the depressive traits screening section [33]. Besides, the instrument aimed to screen the depressive traits, not to diagnose depression.

This scale included eight items from the standard Center for Epidemiological Studies Depression Scale (CES-D). It required participants to rate the frequency of each symptom in the past week from 1 (“little or no (<1 day)”) to 4 (“most of the time (5–7 days)”). The original CES-D was developed by Radloff, which suggested depressive traits could be measured by the four aspects: depressed affect, anhedonia, somatic complaints, and interpersonal problems [35]. The 8-item questionnaire in our study included five items related to depressed affect, two extracted items from the anhedonia sub-scale in the original CES-D, and one item about the somatic symptom. The scores of item 4 and item 6 were reversed in further analyses. (Table 1).

#### 2.3.2. Demographic Variables

In this study, we focused on the following demographic variables: gender (male vs. female) and the region of schools (urban vs. rural area).

### 2.4. Data Analysis

Initially, we would conduct descriptive statistics to describe the sample characteristics. Secondly, we planned to analyse the psychometric properties for the depressive-traits instrument based on the confirmatory factor analysis (CFA). In our CFA model, as there was only one item regarding the somatic symptom, we combined this item with other items related to depressed affect in one dimension. Therefore, theoretically, our CFA model was a two-factor model, including the dimensions of depressed affect and anhedonia. When conducting the CFA model, we adopted the method of “item parcelling” for the dimension of depressed affect in case there were items contributing low factor loadings. This approach has several advantages: (1) enhancing scale commonality, increasing the common-to-unique ratio for each indicator, and reducing the random error, and (2) aggregating scores more approximate to the distribution of the target construct in the comparison of individual items [36]. In this case, we used the single-factor-parcelling method to pair off items with the highest and lowest factor loadings step by step [37]. Due to the limited number of the items, deletion of items with inadequate loadings would decrease the internal consistency significantly. Thus, to avoid sacrificing the internal consistency of the instrument, the approach to dealing with low-factor-loading items was to combine with high-factor-loading items by parcelling method.

Thirdly, we aimed to test the measurement invariance. We constructed three models by setting constraints, respectively. In each model, we separated the whole sample into two groups according to gender or region. The first model, called the configural invariance model, maintained the same two-factor structure for two groups. In the second model for testing the weak invariance, additionally, we kept the factor loadings the same for two groups. In the third model, for examining the strong invariance, one more constraint was set: keep the consistency of intercepts between two groups. Subsequently, the Chi-square difference test was performed to compare the differences between the three models.

Finally, if the CFA model passed the measurement invariance test, we extracted the latent values estimated by depressed affect items and anhedonia items, respectively. Based on the extracted scores, we aimed to investigate the main effects of gender and region and the interaction effect between gender and region in terms of depressed affect and anhedonia by multivariate analysis of variance (MANOVA). All the analyses were performed in RStudio (Boston, MA, USA). For the CFA model, we used the “lavaan” package. For MANOVA analysis, we used the MANCOVA function set in R directly.

## 3. Results

### 3.1. Sample Characteristics

Out of the 2173 surveyed adolescents, 53.2% were males, and 58.4% came from rural areas. The mean age of the participants was 12.40 ± 1.66 (10–15) years, and their overall score of depressive traits was 11.90 ± 3.15 (8–28) (Table 2).

### 3.2. Confirmatory Factor Analysis (CFA) Model

When constructing the CFA model, we initially computed the model without the approach of “parcelling” to see the factor loading of each item. According to the results (See Table 3), we would pair off item 7 and item 3, item 5 and item 2, and item 1 and item 8 as three parcels. The model fit index of the non-parcel model was χ2/df = 111.65.5/19, CFI = 0.969, TLI = 0.955, SRMR = 0.026, RMSEA = 0.047. The correlation between the factor of depressed affect and the factor of anhedonia was 0.35 (Table 3).

Subsequently, we constructed a new CFA model with parcels for the whole sample. In the current study, we adopted the joint model fit criteria based on previous experience. A model should pass four out of five criteria: (1) χ2/df < 5; (2) CFI > 0.9; (3) TLI > 0.9; (4) RMSEA < 0.1; and (5) SRMR < 0.08 [38,39]. The results suggested that the parcel-CFA model reached a good model fit. Moreover, all the factor loadings were above 0.4, which meant all observational variables adequately contributed to the latent variables [40] (Table 4).

### 3.3. Measurement Invariance Tests

We separated the sample into two groups according to gender or region to test if the measurement could apply across them. The configural invariance was examined, and the results suggested the two-factor structure showed a good model fit for four groups (see Table 4). The findings allowed us to continue to test the weak invariance of CFA model. For the male group, we fixed the factor loadings as those in the model for female students. The Chi-square difference test suggested no significant difference between the configural invariance model and the weak invariance model (*p* = 0.70), which supported the weak invariance crossing gender. Following, we fixed the structure, factor loadings, and intercepts in the male group as the female group to test for strong invariance. Again, the Chi-square test result aiming to compare the weak invariance model and the strong invariance was not significant (*p* = 0.99). Thus, the strong invariance crossing the gender was proven. Likewise, the measurement showed weak invariance (*p* = 0.78) and strong invariance crossing region (*p* = 0.99).

### 3.4. Multivariate Analysis of Variance

Based on the parcel-CFA model, we extracted the two latent factor values. The MANOVA analysis was performed for the two dimensions of depressive traits. The multivariate tests results based on Wilks’ lambda criteria suggested the main effects of gender (F = 9.04, *p* < 0.01) and the location of school (F = 3.53, *p* = 0.03). However, the interaction effect between gender and the location was not significant (F = 1.10, *p* = 0.33). The following univariate tests (see Table 5) demonstrated that there was a gender difference in depressed affect (F = 8.69, *p* < 0.01). Besides, the value for anhedonia (F = 4.70, *p* < 0.05) was different across regions of schools (Table 5).

**Figure 1:**
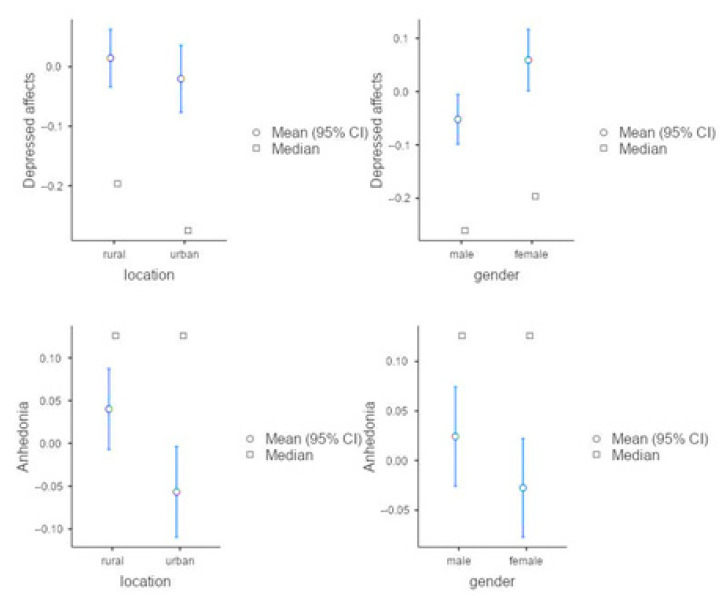
Descriptive Plots of T-tests to Compare the Group Differences in Depressed Affect and Anhedonia among the CFPS-2018 Participants (*n* = 2173).

For interpreting the group difference more clearly, we conducted the T-test. The results suggested that, compared to the boys, girls demonstrated more severe depressed affect (T = 2.97, Cohen’s d = 0.13, *p* < 0.01), and in comparison to urban students, rural students had a higher level of anhedonia (T = 2.65, Cohen’s d = 0.12, *p* < 0.05). However, it is worth noting that the effect sizes of these differences were small (Figure 1).

## 4. Discussion

This study aims to investigate the gender and regional equality in depressive traits among Chinese adolescent students. Our research addresses the gender differences when considering the regional context based on comprehensive national data. As the gender inequality between urban and rural China was significant in the 1990s, it is valuable to probe if it still exists from the mental health perspective in China today.

The main findings are as follows. First, the eight-item scale can be used to screen adolescents’ depressive traits for a national sample in China. Second, compared to the males, female adolescents manifested a higher level of depressed affect, but the effect size of the gender difference is negligible. Third, rural adolescents experience more anhedonia symptoms. However, the distance of anhedonia between the two regions is scant. Fourth, there is no interaction between gender and region in the two dimensions of depressive traits.

Our finding of the higher level of depressed affect for female adolescents is congruent with some previous studies. Except for the physiological reason that females are more vulnerable when facing stressful events because of their levels of cortisol, and so on [14,15], gender intensification is another contributor to the disadvantage faced by girls in regard to mental health. The gender intensification theory noted that when individuals become pubertal, boys will strengthen themselves according to the masculine stereotype, in which males are independent, strong, and lack depression. Moreover, girls will be more strongly linked to the feminine stereotype, in which females are emotional [41,42]. Gender intensification might cause females to display a greater tendency towards depressive characteristics.

Nonetheless, the gender difference in depressed affect is very scant. This finding echoes with cross-cultural research, which indicated that due to the efforts of decades of centralised policies and compulsory education in China, the gender inequality in mental health might be much weaker, even nonexistent. Compared to the USA, the gender difference in terms of depression among adolescents is weaker in China [23].

The rural region is still a potential risk factor for adolescents’ mental health in China. The findings argue that rural adolescents perceive less positive affection. As discussed in Section 1, there is a significant economic gap between rural and urban China. According to the social comparison theory, individuals have the tendency to compare themselves to others in order to acquire knowledge about themselves [43]. At the same time, such a social comparison may have side effects. When individuals are experiencing negative events, such as being in the less advantaged position, the social comparison may decrease their self-esteem and increase the risk of depression [44,45]. Thus, based on the social comparison theory, due to the disadvantaged socioeconomic status (SES) of rural students, when they compare themselves with their peers living in relatively more developed cities, they may be less satisfied with their own less developed living and educational environment, and hence themselves. Eventually, they experience more anhedonia symptoms than urban students. Besides, a meta-analysis proved that, especially in the Asian cultural context where people tend to emphasise educational resources for future generations, the SES is associated with self-value and self-improvement of young people [46].

From the perspective of policy intervention, aiming to promote the development of rural children and adolescents, there have been some programs undertaken. For instance, the Chinese government issued a rural teacher support plan in 2015 to promote the quality of education for every rural child and adolescent [47]. Some local governments are also committed to supporting primary and middle school students in rural areas from miscellaneous aspects, including nutrition, education, psychological service, etc. [48]. In our study, we only found the difference in anhedonia between rural and urban students, which means that, regarding the regional inequality in depressive traits among children and adolescents, we should focus on the positive affect and cognitions rather than depressed aspects, such as self-esteem, life satisfaction, self-efficacy, etc. Thus, we would suggest the government include more positive psychology-related programs to improve young students’ self-esteem and positive self-cognition.

However, the regional difference is not obvious. The phenomenon could be explained by the decreasing social inequality over the years between rural China and urban China. The contributing factors behind this are political efforts. The Chinese government has promoted urbanisation, which helps to improve the economic development and education of rural areas [49]. The findings of the scant effect size of anhedonia and non-significant difference in depressed affect acknowledge the contribution of urbanisation from the perspective of adolescents’ mental health.

No interaction between gender and region in depressive traits was found in our study. Our results are in contrast to the conditions in the 1990s when a number of rural female adolescents demonstrated suicidal ideation because they were treated as the “producers of sons” and faced severe educational deprivation. With urbanisation, a more advanced culture and ideas of gender equality transferred from urban areas to the rural areas, and females no longer play the role of “producers of sons”. Moreover, since 2005, the Chinese government has invested great financial support and human resources in rural areas to guarantee compulsory education for every rural child, as among urban children, especially for the rural children with financial difficulties [50]. Primary-school and middle-school education have been made open and free. Thus, female children’s right to access essential education has been well protected since 2005. Chinese national surveys conducted in the 1980s showed that the illiteracy rate of females (42.27%) was 2.5 times higher than that of males (19.17%). In Beijing, the capital city of China, the advanced education rate of females was only 0.09%. The rate in other regions was from 0% to 0.07% [51]. With the efforts in the implementation of the Compulsory Education Law, the primary school enrollment rate of females reached 99.8%, the same as males in 2014. The female–male ratio in middle school, high school, and university was 46.7%, 50%, and 52.1% in 2014, respectively [52]. All the political efforts made contribute towards rural females facing a much lower risk of mental health problems than in the past. 

## 5. Conclusions

The CFPS-2018 program adopted an applicable national instrument to screen adolescents’ depressive traits. The survey indicated that adolescent gender and regional inequality in terms of mental health is small in China today. However, being female and from a rural area are still potential mental health risk factors. This study suggests that the urbanisation process conducted over decades has contributed to eliminating regional inequality and protecting female children in rural areas. Thus, it should continue. However, we suggest that it is necessary to support children and adolescents in rural areas and females in relation to other aspects. For instance, we should provide additional educational support for rural children to increase their self-esteem. Moreover, it is valuable to provide more psychological service support for female youths. Even though this study found small gender and regional differences in respect of depressive traits, the personal reasons behind them remain unclear. Thus, we recommend further qualitative research to investigate the deeper reasons contributing to the depressive trait differences from an adolescent perspective. There have been various intervention programs aiming to diminish the ongoing rural–urban and male–female inequality. We also suggest it is valuable to track the regional and gender differences in mental health in the future. The evaluation of interventions based on longitudinal research is necessary.

## Figures and Tables

**Table 1:** The 8-item Scale Driven from the CES-D to Screen Individuals’ Depressive Traits.

Item	Item-Aspect
1. I felt depressed	depressed affect
2. I felt that everything I did was an effort	depressed affect
3. My sleep was restless	somatic symptom
4. I was happy	anhedonia
5. I felt lonely	depressed affect
6. I enjoyed life	anhedonia
7. I felt sad	depressed affect
8. I could not get “going”	depressed affect

**Table 2:** Socio-economic Characteristics of the CFPS-2018 Participants (*n* = 2173).

Variable	Outcome	Frequency (*n*)	Percentage (*%*)
Gender	Female	1018	46.8%
	Male	1155	53.2%
Region	Rural	1272	58.4%
	Urban	901	41.5%
Age	10-year-old	377	17.3%
	11-year-old	368	16.9%
	12-year-old	372	17.1%
	13-year-old	383	17.6%
	14-year-old	388	17.9%
	15-year-old	285	13.1%

**Table 3:** Depressive Traits Non-Parcel CFA Model of the CFPS-2018 Participants (*n* = 2173).

Dimension	Item	Factor Loading
Depressed Affect	Item 1	0.602
	Item 2	0.458
	Item 3	0.399
	Item 5	0.656
	Item 7	0.696
	Item 8	0.473
Anhedonia	Item 4	0.698
	Item 6	0.756

**Table 4:** Parcelling CFA Model Fit of the CFPS-2018 Participants’ Depressive Traits (*n* = 2173).

Model	*χ^2^*/*df*	CFI	TLI	SRMR	RMSEA
1. Two-factor structure for the whole sample	19.369/4	0.994	0.984	0.013	0.042
2. Two-factor structure for male students	14.306/4	0.991	0.976	0.018	0.043
3. Two-factor structure for female students	7.561/4	0.997	0.993	0.010	0.030
4. Two-factor structure for urban students	7.685/4	0.996	0.991	0.013	0.032
5. Two-factor structure for rural students	6.33/4	0.994	0.985	0.013	0.040

**Table 5:** Univariate Tests to Probe the Effects of Gender and Region on the CFPS-2018 Participants’ Depressed Affect and Anhedonia (*n* = 2173).

	Dependent Variable	Sum of Squares	Mean Square	F	*p*
Region	Depressed Affect	0.6357	0.6357	0.8385	0.36
	Anhedonia	4.9339	4.9339	7.0116	0.008
Gender	Depressed Affect	6.5892	6.5892	8.6911	0.003
	Anhedonia	1.5826	1.5826	2.249	0.134
Gender × Region	Depressed Affect	0.0473	0.0473	0.0624	0.803
	Anhedonia	1.0315	1.0315	1.4658	0.226
Residuals	Depressed Affect	1644.4269	0.7581		
	Anhedonia	1526.2896	0.7037		

## Data Availability

Publicly available datasets were analysed in this study. This data can be found here: https://opendata.pku.edu.cn/file.xhtml?fileId=10724&datasetVersionId=946 (accessed on 1 August 2021).
